# BRASH Syndrome

**DOI:** 10.1002/ccr3.72833

**Published:** 2026-05-29

**Authors:** Sowdo Nur Iyow, Abdisamad Mohamed Adam, Hassan Adan Ali Adan, Abdulkadir Sağdıç, Ahmed Yusuf Mohamed

**Affiliations:** ^1^ Emergency Medicine Department Mogadishu Somali Türkiye Training and Research Hospital Mogadishu Somalia; ^2^ Internal Medicine Department Mogadishu Somali Türkiye Training and Research Hospital Mogadishu Somalia; ^3^ Internal Medicine Department Eskişehir Şehir Hastanesi Eskişehir Türkiye; ^4^ Research Department Banadir Dialysis Center Mogadishu Somalia

**Keywords:** acute kidney injury, AV nodal blockers, bradycardia, BRASH syndrome, case report, hyperkalemia

## Abstract

BRASH syndrome is a life‐threatening clinical entity characterized by Bradycardia, Renal failure, Atrioventricular (AV) blockade, Shock, and Hyperkalemia. This syndrome is often triggered in patients on AV nodal blocking agents who develop acute kidney injury, leading to a “vicious cycle” of worsening bradycardia and renal dysfunction. We present the case of an 83‐year‐old female with a history of chronic kidney disease and heart failure, who was admitted with severe bradycardia (31 bpm), hyperkalemia (6.9 mg/dL), and shock. Her condition was refractory to standard bradycardia treatment but improved significantly following emergent hemodialysis.

## Key Clinical Message

This case highlights the importance of early recognition of BRASH syndrome and the necessity of aggressive interventions to correct the underlying metabolic abnormalities and reverse the severe hemodynamic compromise.

## 1 Introduction

BRASH syndrome is a recently recognized clinical condition defined by a pentad of five manifestations: **B**radycardia, **R**enal failure, **A**trioventricular (AV) blockade, **S**hock, and **H**yperkalemia [1]. This emerging diagnosis describes the profound bradycardia that occurs in patients taking AV nodal blocking agents who simultaneously present with acute kidney injury (AKI) and hyperkalemia [2].

The syndrome's pathophysiology is believed to originate from a series of events, often triggered by factors like hypovolemia, which worsens renal dysfunction. This leads to the accumulation of both potassium and the AV‐node blocking medications within the patient's system.

Identifying BRASH syndrome is clinically critical because the associated bradycardia can be more severe than anticipated and may be resistant to standard treatment protocols. This resistance can lead to hemodynamic instability and poor patient outcomes [2]. If not treated, this syndrome can lead to catastrophic events. Essential interventions for patient recovery include the rapid institution of hemodynamic support, correction of hyperkalemia, and withdrawal of the beta‐blocker, which may help the patient avoid the need for renal replacement therapy [3].

This report presents the case of an 83‐year‐old female patient whose clinical picture matched BRASH syndrome, and for whom prompt diagnosis and intervention—which included emergent hemodialysis—led to a positive outcome.

## 2 Case History/Examination

An 83‐year‐old female was admitted to the Emergency Department with a lowered level of awareness and sustained bradycardia. Her medical history was significant for diabetes mellitus (DM), stage 4 chronic kidney disease (creatinine clearance of 7.9 mL/min/1.73 m^2^), essential hypertension, and heart failure. Her regular medications included amlodipine (5 mg twice daily) and carvedilol (6.25 mg twice daily). The patient denied experiencing syncope, angina, or palpitations.

Upon presentation, her heart rate was 31 beats per minute, and her blood pressure was 96/43 mmHg, with a mean arterial pressure of 60 mmHg. Her body temperature was 36.4°C. Her respiratory rate was 18 breaths per minute, with 98% peripheral oxygen saturation on room air. The physical examination was otherwise unremarkable.

## 3 Differential Diagnosis, Investigation and Treatment

Key laboratory results included:

- White blood cell count: 5450 cells/μL
- Creatinine: 5.11 mg/dL
- Serum potassium: 6.9 mg/dL
- C‐reactive protein: 93 mg/dL

**FIGURE 1:**
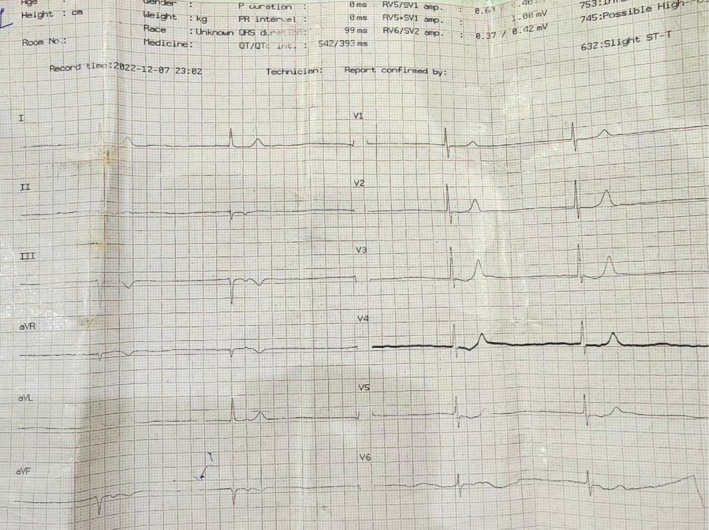
Admission ECG, showing sinus bradycardia with a junctional escape rhythm.

A chest X‐ray was normal. A transthoracic echocardiogram showed normal heart valves and a normal left ventricular dimension, but left ventricular contraction was reduced, with an ejection fraction of 35%–40%. The admission electrocardiogram (ECG) revealed sinus bradycardia with a junctional escape rhythm (Figure 1).

Based on these findings, the hypothesis of BRASH syndrome was considered. The patient's previous medications were suspended. Initial treatment included an intravenous (i.v.) bolus of 200 mL of 0.9% sodium chloride, four 0.5 mg doses of i.v. atropine, 20 mL of 10% calcium gluconate, 80 mL of sodium bicarbonate, and a solution of 150 mL dextrose 20% with 10 U of regular insulin.

Despite these measures, the patient's mean arterial pressure (MAP) remained below 65 mmHg. A continuous infusion of i.v. epinephrine was started and titrated up to a dose of 10 μg/kg/min. The patient was then admitted to the Intensive Care Unit (ICU) for further management.

Due to persistent uremia and hyperkalemia, the patient required emergent hemodialysis, after which the bradycardia improved. The epinephrine infusion was weaned off on day 3. The patient ultimately required long‐term hemodialysis.

## 4 Conclusion and Results (Outcome and Follow‐Up)

**FIGURE 2:**
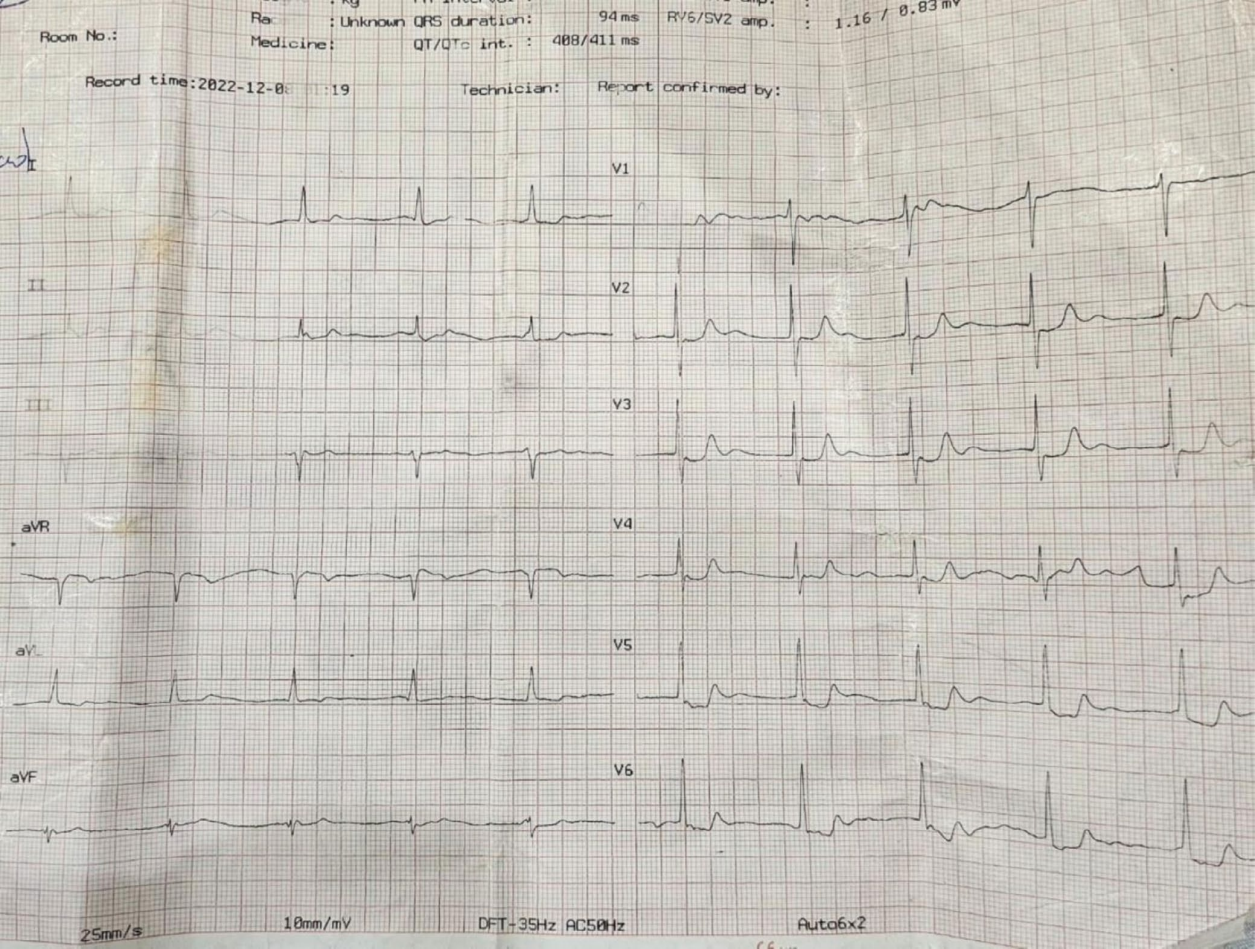
Discharge ECG, showing normal sinus rhythm.

The patient was discharged after 8 days for outpatient cardiology follow‐up, with a recommendation to avoid beta‐blockers. At discharge, her ECG showed normal sinus rhythm (Figure 2), her serum creatinine was 2.36 mg/dL, and her potassium was 4.9 mg/dL (Table 1).

**TABLE 1:** Laboratory results.

Parameter	At admission	Discharge	Reference range
WBC (10^3^/μL)	5.450/μL	4.7 cells/μL	4–10 cells/μL
Creatinine	5.11 mg/dL	2.39 mg/dL	0.5–1.3 mg/dL
Potassium	6.9 mEq/L	4.9 mEq/L	3.5–5.5 mEq/L
C‐reactive protein	93 mg/dL	25 mg/dL	10 mg/dL

Abbreviation: WBC, white blood cells count.

## 5 Discussion

This report describes a rare case of BRASH syndrome, which is characterized by bradycardia, renal failure, AV nodal‐blocking agents, shock, and hyperkalemia [1].

The exact pathophysiological mechanism of BRASH syndrome remains unclear, but it likely involves a synergistic effect between the AV‐nodal blockade and hyperkalemia, which results in severe bradycardia [2, 4]. This bradycardia, in turn, reduces cardiac output, leading to decreased renal perfusion and AKI, which further worsens the hyperkalemia.

A “vicious cycle” is often initiated by hypovolemia or the AV‐nodal‐blocking medications. If not diagnosed quickly, this cycle can progress to shock and multiple‐organ failure, necessitating interventions like transvenous pacing and hemodialysis. Notably, patients with BRASH syndrome are frequently adhering to their proper medication dosing and rarely have supra‐therapeutic (toxic) blood levels of their AV‐nodal‐blocking agents [5].

In this case, the patient was taking carvedilol, a beta‐blocker that is 40% eliminated by the urine; this may have worsened the hyperkalemia and contributed to the spiral of events. The clinical presentation of BRASH syndrome is broad, ranging from asymptomatic bradycardia to full cardiac arrest.

The goals of treatment are based on three main approaches:

1. Correction of hyperkalemia.
2. Hemodynamic support for bradycardia and hypotension.
3. Treatment of any triggering events, such as hypovolemia or stopping AV‐nodal block medication.

In our case, the clinical suspicion of BRASH syndrome was based on the presence of this specific set of signs and the absence of other major diagnoses, like sepsis. Our patient had several conditions associated with a higher risk of developing BRASH syndrome: chronic renal failure, heart failure, and continuous use of a beta‐blocker. Hyperkalemia is also a major component in developing bradyarrhythmia. However, it is interesting to note that our patient's ECG showed significant bradycardia *without* the classic stigmata of hyperkalemia (e.g., peaked T waves or sine waves), a finding that has been reported elsewhere in the literature [6].

The rapid institution of hemodynamic support, correction of hyperkalemia, and beta‐blocker withdrawal was essential to the patient's recovery, though she still required renal replacement therapy. We decided on emergent hemodialysis due to the persistent hyperkalemia, which was followed by the resolution of her bradycardia. In other cases involving large doses of multiple AV node blockers, advanced therapies like lipid emulsion or glucagon could be considered to reverse the AV nodal blockade.

BRASH syndrome is a poorly recognized and potentially lethal clinical entity. While its pathophysiological mechanism is still uncertain, AV‐nodal‐blocking agents and hyperkalemia are known to play a role. Early recognition and treatment of this syndrome are critical to prevent the development of multiple‐organ failure and death.

## Author Contributions

**Sowdo Nur Iyow:** conceptualization, data curation, investigation, methodology, validation, writing – original draft, writing – review and editing. **Abdisamad Mohamed Adam:** conceptualization, data curation, resources, validation, writing – review and editing. **Hassan Adan Ali Adan:** data curation, resources, supervision, visualization, writing – review and editing. **Abdulkadir Sağdıç:** supervision, validation, writing – review and editing. **Ahmed Yusuf Mohamed:** formal analysis, project administration, supervision, writing – review and editing.

## Funding

The authors have nothing to report.

## Ethics Statement

Based on the regulations of the review board of the Mogadishu Somali Turkiye Training and Research Hospital, institutional review board approval is not required for case reports.

## Consent

Written Informed consent was obtained from the patient that her information will be anonymous and will be used only for research purpose.

## Conflicts of Interest

The authors declare no conflicts of interest.

## Data Availability

All original data are available in the Mogadishu Somali Türkiye Training and Research Hospital, Mogadishu, Somalia. We confirm that we bear full responsibility for the data's access and integrity.
